# Interleukin 23 Promotes Hepatocellular Carcinoma Metastasis via NF-Kappa B Induced Matrix Metalloproteinase 9 Expression

**DOI:** 10.1371/journal.pone.0046264

**Published:** 2012-09-25

**Authors:** Jian Li, George Lau, Leilei Chen, Yun-Fei Yuan, Jun Huang, John M. Luk, Dan Xie, Xin-Yuan Guan

**Affiliations:** 1 State Key Laboratory of Oncology in Southern China, Sun Yat-sen University Cancer Center, Guangzhou, China; 2 Department of Clinical Oncology, Li Ka Shing Faculty of Medicine, The University of Hong Kong, Hong Kong, China; 3 State Key Laboratory for Liver Research, Li Ka Shing Faculty of Medicine, The University of Hong Kong, Hong Kong, China; 4 Department of Medicine, Li Ka Shing Faculty of Medicine, The University of Hong Kong, Hong Kong, China; 5 Department of Surgery, Li Ka Shing Faculty of Medicine, The University of Hong Kong, Hong Kong, China; University of North Carolina School of Medicine, United States of America

## Abstract

**Background:**

Hepatocellular carcinoma (HCC) is one of the most popular cancers in the world with poor prognosis, which often develops from chronic liver inflammatory diseases. Interleukin 23 (IL-23) is an inflammatory cytokine which is reported to play an important role in tumor development in animal model. While the function of IL-23 in HCC development remains unknown, so we investigate the role of IL-23 in HCC progression in this study.

**Methodology and Principal Finding:**

Transcript level of IL-23, interleukin17A (IL-17A) and matrix metalloproteinases 9 (MMP9) in clinical HCC samples (n = 81) was determined by qPCR. Protein expression pattern of IL-23 in primary and metastatic HCC tissues pairs (n = 49 pairs) was determined by immunohistochemistry staining. Cell migration, invasion, RNA interfering and immune blotting were used to characterize the functional and signaling mechanisms in IL-23-treated HCC. Compared with paired non-tumor tissue, higher IL-23 expression was detected in HCC tumor tissues with metastasis. Immunohistochemistry staining confirmed the high expression of IL-23 in metastasis HCC. Immune blotting demonstrated that IL-23 was highly expressed in HCC cell lines with metastasis. Functional study found that IL-23 could promote HCC cell migration and invasion. Molecular analysis revealed that IL-23 could upregulate MMP9 expression via NF-κB/p65 signaling activation and IL-17A could improve IL-23 expression in tumor cells directly via activating NF-κB/p65 signaling pathway.

**Conclusions:**

IL-23 could promote HCC metastasis by the upregulation of MMP9 expression via activating NF-κB/p65 signaling pathway. At the same time, IL-17A could further promote IL-23 expression in HCC tumor cells.

## Introduction

Interleukin 23 (IL-23) and interleukin 12 (IL-12) belong to interleukin 6 super-family [1]. IL-12/23 p40 is the common subunit for them, which is covalently linked to either a p19 subunit to form IL-23 or a p35 subunit to form IL-12 [1]. Both cytokines are mainly expressed by activated dendrite cells or macrophages under the stimulation of pathogens. IL-23 was also reported to be secreted by tumor associated macrophages in tumor microenvironment [2].

Interestingly, IL-23 and IL-12 spur different immune pathways [3]. IL-12 induces IFN-γ-producing Th1 cells development and enhances cytotoxic, anti-microbial and anti-tumor responses; whereas IL-23 expands Th17 cells, which is mainly involved in the pathology of autoimmunity and chronic inflammatory disease [4]. Although the role of Th17 in tumor progression remains controversial, the role of IL-23 in tumor incidence and metastasis was established in mouse model. For example, mice lacking IL-23p19 were resistant to DMBA/TPA-induced skin papilloma [5]. In another study, Stat3 signaling was shown to induce IL-23 as well as inhibit IL-12 expression, thereby shifting the balance of tumor immunity toward carcinogenesis [2]. Recently, IL-23 was also reported to promote carcinogenesis and metastasis in the 3′-methylcholanthrene induced fibrosarcoma through suppressing innate immune response [6].

However, the role of IL-23 in HCC progression is poorly explored. In this study, we found that IL-23 was high expressed in HCC tumor cells, especially in those with metastasis. We further proved that IL-23 could directly promote HCC metastasis via NF-kB/relA mediated MMP9 expression. We reported IL-17A was high expressed in HCC with metastasis in another investigation [7], so we further explored the relationship between IL-23 and IL-17A in HCC progression. We found that IL-23 and IL-17A were highly correlated in HCC sample and IL-17A could promote IL-23 expression in HCC cell lines directly.

## Results

### Expression of IL-23 is higher in HCC with metastasis

**Figure 1:**
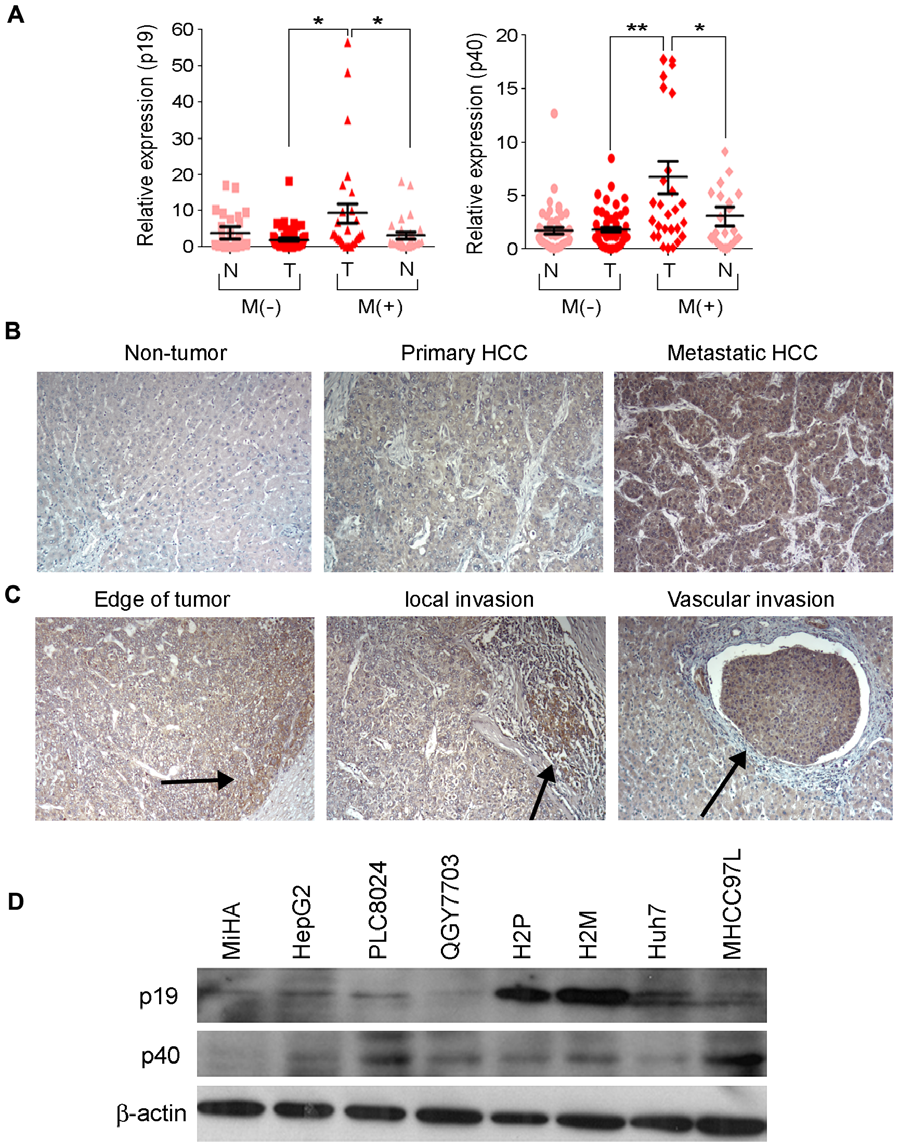
Expression of IL-23 in HCC and HCC cell lines. (**A**) Compare of IL-23 mRNA expression in 28 primary HCC with metastasis (M+) and 53 primary HCC without metastasis (M−) as well as their expression in tumor (T) and non-tumor (N) area. *, P<0.05; **, P<0.01. (**B**) Representative of IL-23 expression in non-tumor, primary HCC and metastasis HCC by IHC staining (magnification 200×). (**C**) Representative of IL-23 expression in HCC by IHC staining (magnification 200×). (**D**) Protein expression of IL-23 in HCC cell lines detected by western blot. β–actin was used as loading control.

IL-23 mRNA expression in 81 paired HCC tissue demonstrated that no significant difference of IL-23p19 expression was detected between tumors and their non-tumor counterparts. However, expression of IL-23p40 in tumors was significantly higher than that in their non-tumor counterparts (*P* = 0.003, paired-samples T test). As IL-23 has been reported to be associated with tumor metastasis in animal model, the expression of IL-23 was further characterized in HCC with or without metastasis. The results showed that both subunits of IL-23 were significantly higher expressed in tumor area than that in non-tumor counterparts (for p19, *P* = 0.035; for p40, *P* = 0.027, paired-samples T test) (Fig. 1A) in 28 HCC with metastasis. While no significant different expression was observed between tumors and their non tumor counterparts (*P* = 0.265 for p19 and *P* = 0.754 for p40, paired-samples T test) (Fig. 1A) in 53 HCC without metastasis.

Association study was further applied to investigate the clinical significance of IL-23 expression in 81 HCC cases. The results showed that tumor area IL-23 expression did not correlate to patients' age, gender, cirrhosis status, TNM stage and tumor size, while tumor area IL-23 (p19 and p40) expression was significantly correlated to patients' metastasis status (Table 1). The result suggested that IL-23 might play an important role in HCC metastasis.

**Table 1:** Correlation of IL-23 expression^1^ with clinicopathological features in 81 HCC patients.

Clinicopathological Features	Number (n = 81)	p19 (Mean ±SD)^1^	P value	p40 (Mean ±SD)^1^	P value
***Gender***			0.384		0.229
Female	17	6.52±4.67		5.54±3.98	
Male	64	4.19±2.37		2.98±1.07	
***Age***			0.801		0.066
≤50	46	4.44±2.69		4.39±1.94	
>50	35	4.99±3.43		2.37±0.97	
***HBsAg*** 2			**0.046**		0.085
Negative	19	10.28±6.55		6.18±3.65	
Positive	59	3.05±1.8		2.73±1.07	
***Cirrhosis*** 2			0.297		0.392
Absent	25	6.64±4.62		4.42±2.68	
Present	54	3.91±2.33		3.13±1.29	
***Serum AFP (ng/ml)*** 2			0.667		0.742
≤25	22	4.21±2.22		3.19±2.97	
>25	55	5.01±2.99		3.73±1.31	
***Tumor size (cm)*** 2			0.603		0.071
≤5	34	4.06±3.39		2.34±1.1	
>5	46	5.2±2.77		4.35±1.94	
***Tumor multiplicity*** 2			0.701		0.577
Solitary	56	4.46±2.5		2.91±1	
Multiple	22	5.46±4.54		3.87±3.24	
***TNM stage*** 2			0.050		0.141
I–II	55	2.63±0.97		2.73±1.02	
III–IV	24	9.37±6.48		5.28±3.21	
***Metastasis***			**0.014**		**0.003**
No	53	2.14±0.85		1.82±0.48	
Yes	28	9.49±5.52		6.72±3.01	

1 Expressions of p19 and p40 were detected by qPCR. Values are expressed as mean±SD.

2 Partial data unavailable, statistics was done on the available data. Difference is considered significant when *P*<0.05 (shown in bold).

### IL-23 is predominantly expressed in tumor cells

Protein expression of IL-23 was further detected by IHC staining in 49 paired primary and metastatic HCC specimens. IL-23 was predominantly detected in tumor cells with metastasis (Fig. 1B). Interestingly, increasing IL-23 expression was often observed in edge area, invading area, or blood vessel invading area tumor cells (Fig. 1C). Among them, 28 pairs (57.1%) showed higher, 2 pair (4.08%) showed weaker expression of IL-23 in metastatic HCC than their respectively matched primary tumor samples. 17 pairs (27.27%) showed the same expression in primary and metastasis samples. And 2 pairs (4.08%) of them showed negative expression.

Immune blotting was applied to determine protein expression level of IL-23 in one immortalized liver cell lines (MiHA) and seven HCC cell lines (HepG2, PLC8024, QGY7703, H2P, H2M, Huh7 and MHCC-97L). The result demonstrated that both subunits of IL-23 could be detected in all HCC cell lines (Fig. 1D).

### RhIL-23 increases HCC cells motility

**Figure 2:**
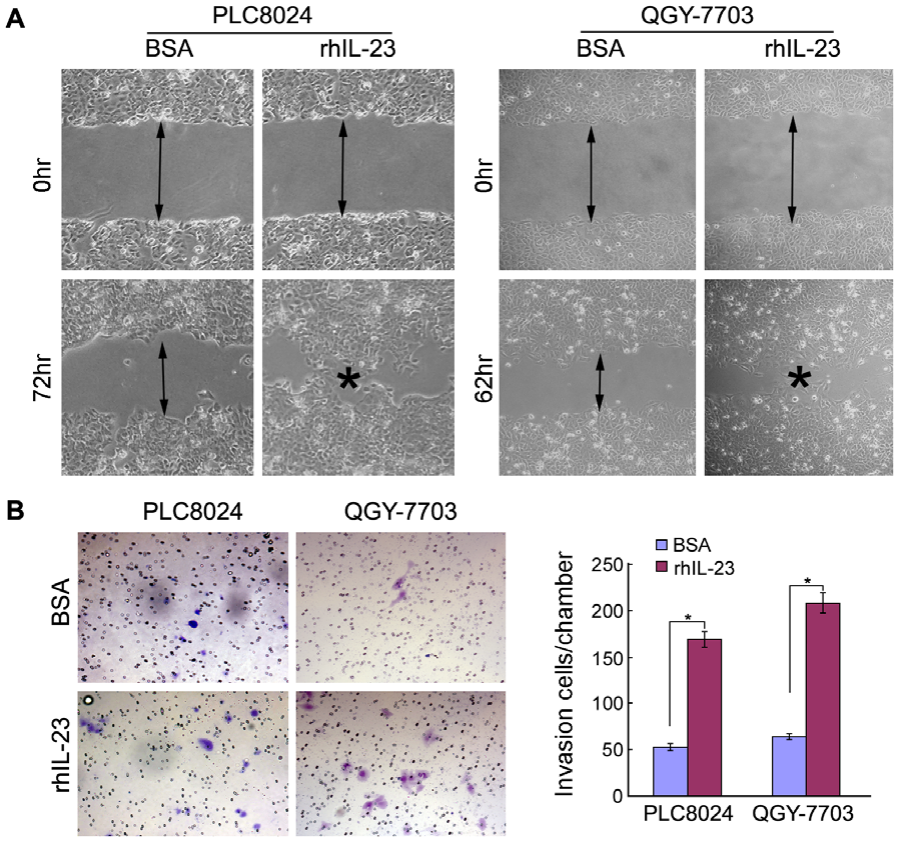
IL-23 promotes HCC cell migration and invasion. (**A**) RhIL-23 treated HCC cells (PLC8024 and QGY-7703) showed higher motility in wound-healing assay, compared with BSA buffer control treated cells. (**B**) RhIL-23 increased cell invasion as detected by cell invasive assay. Representatives of cells migrated through Matrigel-coated transwell were shown in the left panel (magnification 100). Total invasive cell number in each chamber was summarized in the right panel. *, *P*<0.05.

Wound healing and matrigel invasion assays were performed to detect the effect of IL-23 on tumor cells motility as IL-23 expression level was proved to be associated with tumor metastasis. The results demonstrated that rhIL-23 could remarkably promote cell migration and invasion comparing to control parental cells (Fig. 2A and 2B).

### Knocking down IL-23 expression by RNAi inhibits cells motility

**Figure 3:**
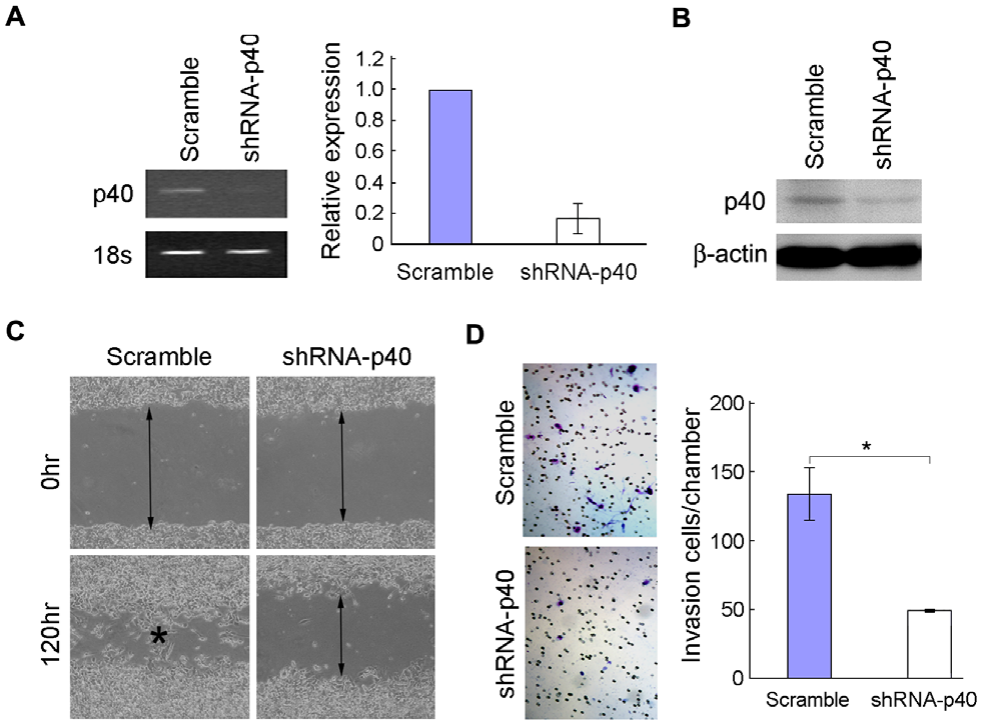
Knocking down IL-23 expression inhibits cell motility. (**A**), (**B**) IL-23 p40 expression was efficiently silenced by IL-23p40 shRNA as determined by RT-PCR, qPCR (**A**) and western blot (**B**) in MHCC-97L cell. 18S and β-actin were used as loading control respectively. (**C**) and (**D**) Compared to vector control the migration (**C**) and invasion (**D**) ability of IL-23 silenced cells were greatly decreased in MHCC-97L cell. *, P<0.05.

To test whether the endogenous IL-23 is important for cancer cell motility or not, we knocked down IL-23 expression in HCC cells. As p40 expression is the restriction step for IL-23 expression [1], Short hairpin RNA (shRNA) against *IL-23p40* gene was used to silence IL-23 expression in MHCC-97L cells. The result showed that the endogenous expression of IL-23p40 could be efficiently silenced in MHCC-97L cells at both mRNA (Fig. 3A) and protein levels (Fig. 3B). Silencing IL-23p40 could effectively knock down the endogenous expression of IL-23 in MHCC-97L, but had little effect on the expression of IL-12 (data were not shown). Functional study demonstrated that knocking down IL-23 expression could significantly inhibit the wound healing (Fig. 3C) and invasive abilities of MHCC-97L cells (Fig. 3D).

### RhIL-23 up regulates MMP9 expression in HCC cells

**Figure 4:**
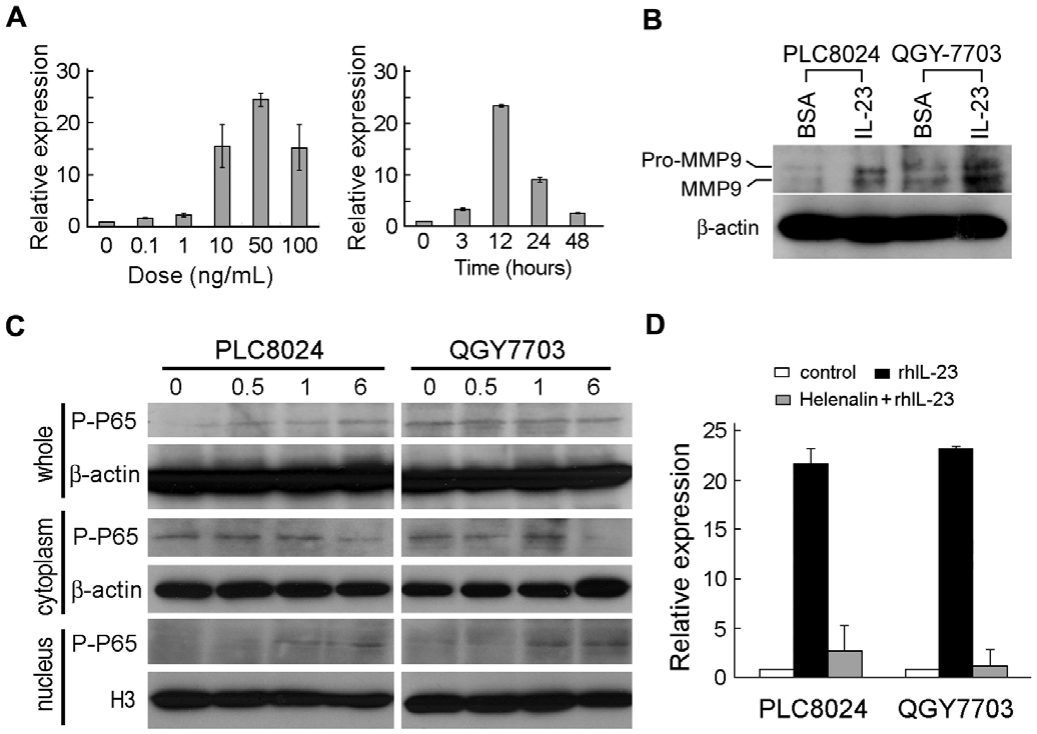
IL-23 upregulates MMP9 expression via activating NF-κB. (**A**) IL-23 promoted MMP9 mRNA expression at a dose and time dependent manner shown by qPCR. (**B**) Expression of MMP9 was detected by western blot analysis in HCC cells treated with rhIL-23 (50 ng/mL) or BSA buffer control for 24 hours. (**C**) Western blot analysis was used to detect whole cell, cytoplasm and nucleus level of P-P65 (active form of NF-κB) expression in PLC8024 and QGY-7703 cells treated with rhIL-23 (50 ng/mL) at indicated time. (**D**) Expression of MMP9 detected by qPCR in PLC8024 and QGY-7703 cells with different treatment. BSA: treated with BSA buffer control; rhIL-23: treated with rhIL-23 (50 ng/mL); Helenalin+rhIL-23: treated with helenalin (0.l µM) and rhIL-23 (50 ng/mL). *, *P*<0.05.

As IL-23p19 knockout mice were reported to have decreased MMP9 expression [5], which played an important role in tumor metastasis [8], we studied whether IL-23 influenced HCC MMP9 expression or not. The result demonstrated that IL-23 increased MMP9 mRNA expression in a dose dependent manner and reached the peak after treatment for 12 hr (Fig. 4A). Immune blotting assay also confirmed that rhIL-23 could increase MMP9 expression at protein level (Fig. 4B). IL-23 had no effect on the expression of other MMPs including MMP1, MMP2, MMP3, and MMP10 (data were not shown).

### RhIL-23 up regulates MMP9 expression via activating NF-κB/P65

As NF-κB is the key transcript factor for MMP9 expression [9], we next investigated whether NF-κB also involved in IL-23 induced MMP9 expression or not. The result demonstrated that the active form of NF-κB (P-P65) in cytoplasm was significantly decreased while the level of which in nucleus was dramatically elevated in PLC8024 and QGY-7703 cells after rhIL-23 treatment for 6 hr (Fig. 4C). When helenalin, a NF-κB inhibitor, was added to PLC8024 and QYG-7703 medium1 hr before rhIL-23 treatment, MMP9 mRNA expression was significantly decreased (*P*<0.05, Independent Student's *t*-test) (Fig. 4D). The result proved that IL-23 increased MMP9 expression in HCC cells was dependent on NF-κB/P65 activation.

### RhIL-17A can promote IL-23 expression in HCC cell lines via activating NF-κB/P65

**Figure 5:**
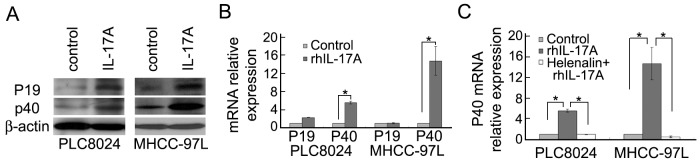
IL-17A promots IL-23expression in HCC. (**A**) Expression of IL-23 was detected by western blot analysis in HCC cells treated with or without rhIL-17A (50 ng/mL) for 24 hours. (**B**) Transcriptional expressions of both subunits of IL-23 were compared by qPCR between cells treated with and without rhIL-17A (50 ng/mL) for 12 hours. *, *P*<0.05. (**C**) Transcriptional expression of IL-23p40 was detected by qPCR in PLC8024 and MHCC-97L cells with different treatment. Control: without rhIL-17A treatment; rhIL-17A: treated with rhIL-17A (50 ng/mL); Helenalin+rhIL-17A: treated with helenalin (0.l µM) and rhIL-17A (50 ng/mL). *, *P*<0.05.

As IL-17A and IL-23 are frequently reported to coexist in many diseases [10], and IL-17A is also reported to promote HCC metastasis [7], we further investigate the relationship between IL-23 and IL-17A in HCC. We found that rhIL-17A (50 ng/mL) could significantly increase the expression of IL-23 in PLC8024 and MHCC-97L cell lines at protein level (Fig. 5A). Further investigation showed that rhIL-17A could significantly increase the transcription level of IL-23p40 in HCC cell lines (PLC8024 and MHCC-97L) (*P*<0.05), but had little influence on the transcription expression of IL-23p19 (Fig. 5B). IL-17A was reported to promote the NF-κB activation in HCC cell lines [7]. We next verified whether the up-regulating effect of IL-17A on IL-23 expressions was also via the activation of NF-κB or not. When helenalin, a NF-κB inhibitor, was added to PLC8024 and MHCC-97L medium before rhIL-17A treatment, IL-23p40 mRNA expression was significantly decreased (*P*<0.05, Independent Student's *t*-test) (Fig. 5C), which demonstrated that IL-17A induced IL-23 expression in HCC cells was via NF-κB/P65 activation.

### IL-23 is positively correlated with expression of MMP9 and IL-17A in clinical samples

**Figure 6:**
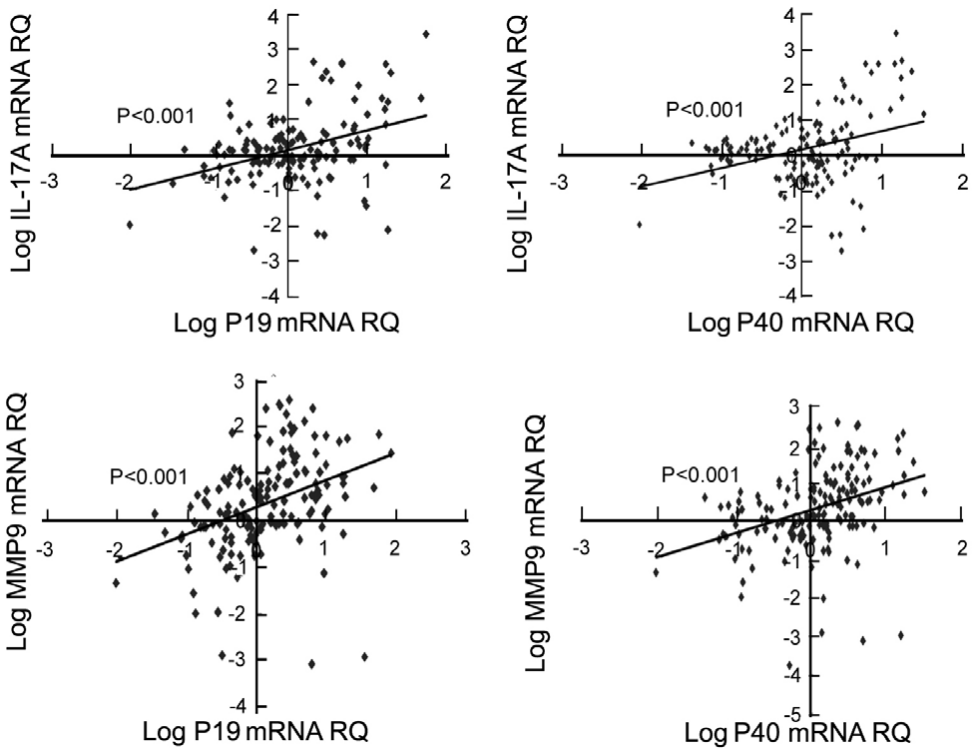
IL-23 correlates with IL-17A and MMP9 expression in HCC clinical samples. Expression of IL-23 was positively associated with IL-17A and MMP9 expressions in 81 pair clinical HCC specimens (shown only by sample that have been detected). Analyzed with linear regression lines and pearson correlation by SPSS16.0.

qPCR result of MMP9, IL-17A and IL-23 mRNA expression in 81 clinical HCC specimens were further analyzed with SPSS16.0 software to confirm the correlation of MMP9, IL-17A and IL-23. The results demonstrated that the mRNA expression of IL-23p19 was significantly correlated with expressions of IL-17A (R = 0.36, *P*<0.001) and MMP9 (R = 0.411, *P*<0.001) (Fig. 6) the expression of IL-23p40 also significantly correlated with expression of IL-17A (R = 0.337, *P*<0.001) and MMP9 (R = 0.349, *P*<0.001) (Fig. 6) in clinical HCC samples.

## Discussion

Recent investigations proved that IL-23 played an important role in tumorigenesis through its immune suppression function [2], [5]. Here we found for the first time that IL-23 was high expressed in HCC with metastasis. Invasion of the extracellular matrix is the first step for tumor metastasis. To verify whether the high expression of IL-23 in metastasis tumor cells was only the marker for them or it could also promote tumor invasion and migration, we treated HCC cell lines with rhIL-23 as well as knocked down the endogenous IL-23 expression by RNAi. Results demonstrated that IL-23 was not only the marker for metastatic tumor cells, it could also promote tumor cells invasion and migration directly, which implied that IL-23 was a potential biomarker as well as a functional marker for metastasis HCC.

Twist mediated morphology change [11] and snail induced EMT [12] are important events in tumor invasion and metastasis, we did not observe significantly EMT change after rhIL-23 treatment (data were not shown). As increased MMPs expressions are also reported to advance tumor metastasis [8], [13], we followed up to study whether IL-23 could influence MMPs expression or not. The result verified that IL-23 could up regulate MMP9 expression in HCC cell lines. Data from clinical samples also proved that MMP9 expression was highly (*P*<0.001) correlated with IL-23 expression. As NF-κB is the key transcript factor for MMP9 expression [9], we further investigated whether IL-23 could activate NF-κB signaling pathway or not. The result proved that rhIL-23 could activate NF-κB through promoting the nucleus translocation of P-P65. Further study proved that IL-23 increased MMP9 expression could be efficiently inhibited by NF-κB inhibitor, which suggested that IL-23 increased MMP9 expression was via the activation of NF-κB/p-p65 transcription factor.

In our recent study, IL-17A was found to high expressed in HCC and can promote the metastasis of HCC [7]. As Il-23 and IL-17A are frequently correlated in many diseases [10], we further studied the correlation of IL-17A and IL-23 in HCC. It was demonstrated that IL-17A could increase the expression of IL-23 in HCC tumor cells through promoting the transcriptional expression of IL-23p40, which is the restriction expression subunit for IL-23 expression. Clinical results from HCC samples further confirmed the positive correlation of IL-23 and IL-17A.

Tumor metastasis is a multistage event, in which multiple factors are involved. It requires the cancer cells to escape from the primary tumor, survive in the circulation, seed at distant site and grow. Each process is determined by the tumor cells as well as the local tumor microenvironment. Tumor cells preserve the invasion and migration ability is the admission for it. Then it may also need the local tumor microenvironment to provide a permission status for tumor cells to survive and even a status that could promote the metastasis of malignant cells. High expression of IL-23 could help to facilitate tumor metastasis in many aspects. Tumor cells with high expression of IL-23 have higher invasion and migration ability than their peers that have lower expression, which was verified in the current study. The expression of IL-23 in tumor microenvironment was reported to can attract macrophages [5], which were reported to be obligated partners for tumor metastasis [14]. In our study, the number of macrophages was significantly increased around the high IL-23 expressed area (data were not shown). IL-23 was also reported to act on Treg cells to suppress the function of CD8 T cells in local tumor microenvironment [2]. Treg cells had been reported to locate in HCC local microenvironment and the increased frequency of which was correlated with CD8 T-cell impairment and poor survival of HCC patients [15].

## Materials and Methods

### HCC samples and cell lines

HCC specimens for mRNA were obtained from 81 patients underwent hepatectomy in the Sun Yat-Sen University Cancer Center (Guangzhou, China). Among them, 28 with metastasis including 8 intra-hepatic, 11 portal vein, and 9 extra-hepatic metastasis (2 in gall bladder, 2 in greater omentum, 1 in lymph node, 2 in colon and 2 in diaphragma). 49 pairs of primary and matched metastatic HCC specimens were collected from archives of paraffin embedded tissues in the Sun Yat-Sen University Cancer Center, which included 32 intrahepatic (29 in portal vein and 3 in intercostal vein) and 17 extrahepatic metastasis (2 in greater omentum, 6 in lymph nodes, 3 in abdominal cavity, 1 in inferior vena cava, 1 in adrenal cortex, 2 in peritoneum, 1 in kidney and 1 in bone). Informed written consent was obtained from each patient included in the study and the study protocol conforms to the ethical guidelines of the 1975 Declaration of Helsinki as reflected in a priori approval by the Committees for Ethical Review of Research involving Human Subjects at Sun Yat-Sen University Cancer Center. All used human HCC cell lines was acquired and maintained as previously described [7], [16].

### Immunohistochemistry (IHC) staining

IHC staining was performed as previously described [7]. Briefly, after blocking with normal goat serum or 5% BSA in PBS, The slides were incubated with primary antibody against IL-23 P19 (mouse anti-IL-23 p19 diluted with 1∶100, Biolegend, Minneapolis, MN or rabbit anti-IL-23 P19 diluted with 1∶500, Abcam, UK) at 4°C overnight in a moist chamber. Then followed up with second antibody binding and detected with Diaminobenzidine tetrahydrochloride. To evaluate the IHC staining of IL-23p19, expression of IL-23p19 was scored as negative, weak, moderate, and strong as previously described [16]. Positively stained cells were scored by two independently investigators under microscope.

### Cell migration and invasion assay

Cell migration and invasion assay were performed as described previously [7]. Briefly, cells were treated either with 50 ng/mL recombinant human IL-23 (rhIL-23) (R&D System Minneapolis, MN) or BSA buffer control for described time and observed accordingly.

### RNA interference (RNAi) assay

MHCC-97L cells were transfected with a pGFP-RS plasmid encoding IL-23p40 shRNA (Origene, Rockville MD) or a scramble control pGFP-V-RS using Lipofectamine 2000 reagent (Invitrogen, Carlsbad, CA) according to the manufacturer's instruction. Gene silencing efficiency was measured by real time PCR, quantitative real-time PCR (qPCR) and western blotting after stable pour selection with 1 ug/mL puromycin (Sigma-Aldrich, St. Louis, MO).

### Immune blotting

Whole cell lysates, Cytoplasm and Nuclear lysates were isolated as described previously [7]and immune blotting were performed with the standard protocol with antibodies against hIL-12P40 (abD Serotec, UK), β-actin, P-p65 (Santa Cruz Biotechnology, Santa Cruz, CA), IL-17A (R&D Systems, Minneapolis, MN), MMP9, histone H3 and hIL-23p19 (Abcam, UK).

### qPCR

Total RNA extracting, cDNA amplifying and quantifying as described previously [7]. The followings were primers used in this investigation: IL-23P19 (Fw: 5′-GGACAACAGTCAGTTCTGCTT-3′; Rv: 5′-CACAGGGCTATCAGGG AGC-3′); IL-23p40: (Fw: 5′-ATGGTGAGCCGTGATTGTGC; Rv 5′- AGGGAT TCCAGATTTTCTTTGC-3′); 18 s: (5′-CTCTTAGCTGAGTGTCCCGC-3′, Rv: 5′-CTGATCGTCTTCGAACCTCC-3′; MMP9: (Fw: 5′-GGGACGCAGACATCGT CATC-3′; Rv: 5′-TCGTCATCGTCGAAATGGGC-3′); IL-17A: (Fw: 5′- CAATCCC ACGAAATCCAGGATG-3′; Rv: 5′- GTGGAGATTCCAAGGTGAGG -3′).

### Statistical analysis

All data were analyzed with SPSS software (version 16.0). Comparisons between groups were analyzed by Student's t-test. The two-tailed chi-squared test was used to analyze the association of IL-23 expression with HCC metastasis status. Correlation between variables was determined by linear regression analysis. Value of *P*<0.05 (two-tailed) was considered statistically significant.
